# Treatment of a Prolonged Air Leak with Radiotherapy: A Case Report

**DOI:** 10.1155/2012/158371

**Published:** 2012-09-27

**Authors:** Erdoğan Çetinkaya, M. Akif Özgül, Şule Gül, Ertan Çam, Yakup Büyükpolat

**Affiliations:** ^1^Yedikule Chest Diseases and Thoracic Surgery Education and Research Hospital, Chest Diseases Department, 34020 Istanbul, Turkey; ^2^Okmeydani Education and Research Hospital, Radiation Oncology Department, 34020 Istanbul, Turkey

## Abstract

Pneumothorax is defined as air in the pleural space. Depending on the severity of the pneumothorax, treatment consists of oxygen therapy, simple aspiration, tube thoracostomy, and pleurodesis. Prolonged air leakage is observed in 25% of the patients who have undergone surgical procedures, such as thoracotomy, pleurectomy, and video-assisted thoracoscopy. The patient presented here is the third reported case successfully treated with radiotherapy. Ventilation scintigraphy was used to localise the air leak, and localised radiotherapy was performed at the targeted location. After radiotherapy, the air leak ceased and at the 3-month followup, the pneumothorax had not recurred. Radiotherapy can be a treatment modality for patients with prolonged air leak, who are not candidates for surgery.

## 1. Introduction 

Prolonged air leakage is observed in 25% of the patients who have undergone surgical procedures. In patients who are not candidates for surgical treatment, alternative methods are used to treat the prolonged air leak. We present a case who has air leakage for 1 month and successfully treated with radiotherapy. 

## 2. Case

A 77-year-old male patient was admitted to the intensive care for respiratory failure and a pneumothorax was identified; tube thoracostomy was performed. The patient's general condition improved and he was extubated and discharged after ending the tube thoracostomy. The patient was readmitted 15 days later due to shortness of breath, and right recurrent pneumothorax was identified. The tube thoracostomy was repeated. After performing negative aspiration and talc pleurodesis twice, the patient's drainage ended on day 14. The patient was readmitted 15 days later due to shortness of breath. In his history, he had smoked cigarettes for 60 pack/years and had been treated for chronic obstructive pulmonary disease (COPD) for 10 years. 

**Figure 1:**
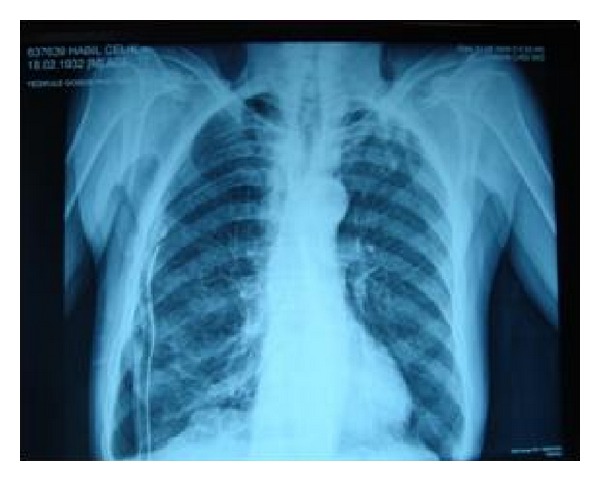
Chest radiography: a total pneumothorax in the right lung.

**Figure 2:**
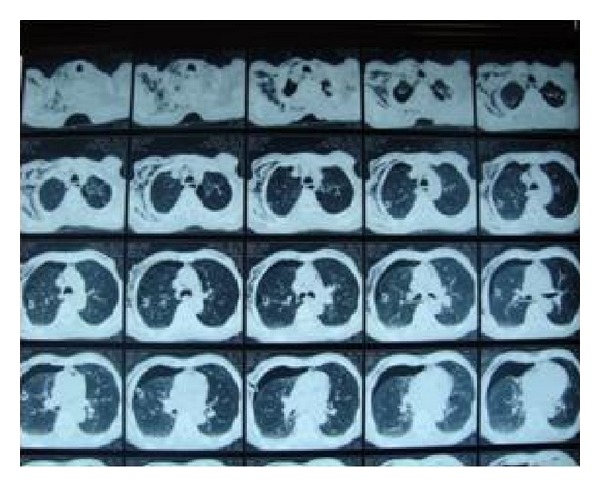
Thorax CT: bullous areas at the apexes of both lungs and a right pneumothorax.

A posteroanterior (PA) chest X-ray showed bilaterally increased emphysematous ventilation in the lung parenchyma and a total pneumothorax in the right lung (Figure 1). On thoracic-computed tomography (CT), bullous areas at the apexes of both lungs and a right pneumothorax were observed (Figure 2). 

**Figure 3:**
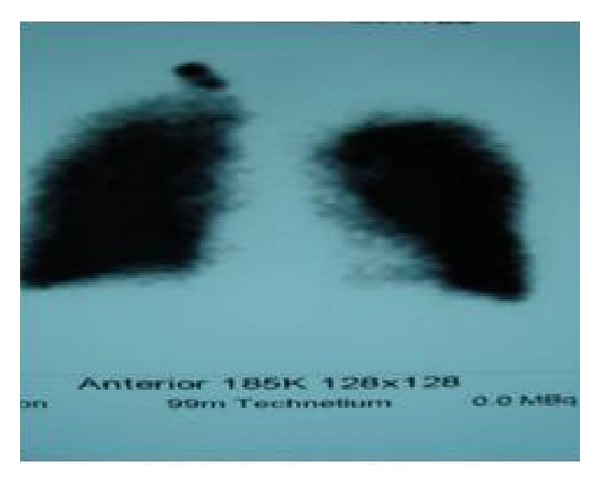
Ventilation scintigraphy: refractory air leak in the right upper lobe.

**Figure 4:**
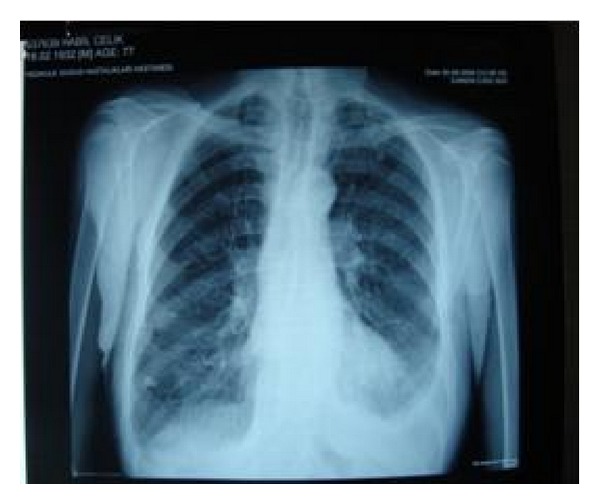
Chest radiography after treatment.

Surgery was considered, but was contraindicated by the patient's advanced age and comorbidities (cardiac failure and severe COPD). Based on a published report, radiotherapy treatment was attempted. Ventilation scintigraphy showed findings consistent with the refractory air leak in the right upper lobe (Figure 3). Local radiotherapy at 10 Gy was applied to a 10 × 10 cm area determined using ventilation scintigraphy with a 3 cm margin, twice, 1 week apart. A followup chest X-ray showed complete expansion of the lung and no air leak. The tube thoracostomy was ended. No pneumothorax was observed at the 3-month followup (Figure 4). 

## 3. Discussion 

Primary pneumothorax is seen in people who do not have any underlying disease. Secondary pneumothorax develops as a result of an underlying lung disease [1]. The clinical effects of pneumothorax depend on the reduction in the arterial oxygen level and vital capacity. Thus, the main goals of treatment are to reexpand the collapsed lung, eliminate the symptoms, and prevent recurrences [2].

The most important point to consider in treating pneumothorax is the patient's symptoms and the diameter of the pneumothorax. Patients who are asymptomatic and have a pneumothorax less than 2 cm can be followed using oxygen treatment. Those who have a pneumothorax that is less than 2 cm, yet have symptoms, such as shortness of breath, can initially be treated with simple aspiration. In patients who do not respond to these treatments or have a pneumothorax larger than 2 cm and are symptomatic, the initial treatment method is tube thoracostomy. In difficult or recurrent pneumothoraces, pleurodesis can be performed. In patients whose lungs do not expand or have a prolonged air leak despite treatment, pleurectomy or surgery, such as VATS, can be performed [3]. With a prolonged air leak lasting for more than 7 days, surgical methods are recommended [4]. In a series of 115 cases, Chee et al. stated that in 75% of the cases the air leak disappeared completely after 7 days and in 97% it disappeared after 14 days; thus, they recommended surgical treatment after 14 days [5]. Especially in patients with underlying lung disease, a higher rate of postoperative complications has been reported after surgery was performed due to a prolonged air leak. Thomas et al. reported a rate of 14.9% for secondary spontaneous pneumothorax versus 7.2% for primary spontaneous pneumothorax [6]. 

In patients who are not candidates for surgical treatment, alternative methods are used to treat the prolonged air leak. One of these is endobronchial valve implementation with flexible bronchoscopy. In a 2009 review by Travaline et al. [7], in 40 patients who had an endobronchial valve placed because of a prolonged air leak, the air leakage disappeared completely in 47.5% and was reduced in another 45%. 

Ong et al. [8] considered surgical treatment for a patient who had bullous lung disease and had a tube thoracostomy, which did not result in expansion, with prolonged air leak for 4 weeks. However, the patient was deemed unfit for surgery. As a result, the location of the air leak was determined with ventilation scintigraphy, and radiotherapy was administered to this area to induce fibrosis. No air leak was detected in follow-up ventilation scintigraphy performed approximately 3 weeks after the initial fraction. 

There is also another case report in which local radiotherapy was used in a patient with a persistent pneumothorax unsuccessfully treated with apical bullectomy and fibrin glue application at thoracoscopy [9]. 

Thorax radiotherapy is a known treatment method, which has been used for many years, to increase the success of local treatment in lung cancer. Its most important dose-limiting effect is radiation fibrosis. While the effective threshold dose for microscopic tumours is 50 Gy, the threshold dose for maintaining the lung is 30 Gy. Following cell death due to radiation, scar tissue or fibrosis forms. Tissue repair leads to the release of biological mediators, which, in turn, increases fibroblast migration, leading to collagen accumulation and fibrosis [10]. 

Our patient had a history of recurrent pneumothorax, bilateral bullous areas on thorax CT, and his air leakage had continued for 1 month. Initially, surgical treatment was considered, but it was contraindicated by his advanced age and comorbidities. Based on a report, the air leak was located using ventilation scintigraphy and local radiotherapy was applied to this area. In a follow-up chest X-ray taken 3 weeks later, the lung was found to be expanded completely and the air leak had stopped. Consequently, the tube thoracostomy was removed and no pneumothorax was observed at the 3-month followup. 

We believe that radiotherapy is another treatment option for a prolonged air leak in patients with underlying lung disease and for whom surgery is too risky. However, it is necessary to observe treatment results from many more cases to validate this treatment.
